# Prostate specific membrane antigen (PSMA) expression in primary gliomas and breast cancer brain metastases

**DOI:** 10.1186/1475-2867-14-26

**Published:** 2014-03-20

**Authors:** Natsuko Nomura, Sandra Pastorino, Pengfei Jiang, Gage Lambert, John R Crawford, Marco Gymnopoulos, David Piccioni, Tiffany Juarez, Sandeep C Pingle, Milan Makale, Santosh Kesari

**Affiliations:** 1 Translational Neuro-Oncology Laboratories, Moores Cancer Center, UC San Diego, 3855 Health Sciences Drive, MC#0819, La Jolla, CA 92093-0819, USA; 2 University of Hawaii cancer Center, 701 ILALO Street, Bldg A-4R, Rm 451, Honolulu HI 96813, USA; 3 Department of Neurosciences, UC San Diego, La Jolla, CA, USA; 4 Pediatric Neurology, Rady Children’s Hospital, San Diego, CA, USA; 5 Ambrx Inc, La Jolla, CA 92037, USA

**Keywords:** PSMA, VWF, Gliomas, Brain metastases, Targeted therapy, Breast cancer

## Abstract

**Background:**

Primary and secondary brain cancers are highly treatment resistant, and their marked angiogenesis attracts interest as a potential therapeutic target. Recent observations reveal that the microvascular endothelium of primary high-grade gliomas expresses prostate specific membrane antigen (PSMA). Breast cancers express PSMA and they frequently form secondary brain tumors. Hence we report here our pilot study addressing the feasibility of PSMA targeting in brain and metastatic breast tumors, by examining PSMA levels in all glioma grades (19 patients) and in breast cancer brain metastases (5 patients).

**Methods:**

Tumor specimens were acquired from archival material and normal brain tissues from autopsies. Tissue were stained and probed for PSMA, and the expression levels imaged and quantified using automated hardware and software. PSMA staining intensities of glioma subtypes, breast tumors, and breast tumor brain metastases were compared statistically versus normals.

**Results:**

Normal brain microvessels (4 autopsies) did not stain for PSMA, while a small proportion (<5%) of healthy neurons stained, and were surrounded by an intact blood brain barrier. Tumor microvessels of the highly angiogenic grade IV gliomas showed intense PSMA staining which varied between patients and was significantly higher (p < 0.05) than normal brain. Grade I gliomas showed moderate vessel staining, while grade II and III gliomas had no vessel staining, but a few (<2%) of the tumor cells stained. Both primary breast cancer tissues and the associated brain metastases exhibited vascular PSMA staining, although the intensity of staining was generally less for the metastatic lesions.

**Conclusions:**

Our results align with and extend previous data showing PSMA expression in blood vessels of gliomas and breast cancer brain metastases. These results provide a rationale for more comprehensive studies to explore PSMA targeted agents for treating secondary brain tumors with PSMA expressing vasculature. Moreover, given that PSMA participates in angiogenesis, cell signaling, tumor survival, and invasion, characterizing its expression may help guide later investigations of the poorly understood process of low grade glioma progression to glioblastoma.

## Background

Primary and secondary brain cancers are difficult to treat and are a major cause of cancer-related death [1-3]. Gliomas are the most common primary brain tumors, with grades I, II, and III usually progressing to a poor outcome over 2 to 10 years, while the aggressive grade IV (glioblastoma; GBM) advances very rapidly within 2–3 years, causing approximately 13,000 deaths per year [1]. Secondary brain cancers occur in an estimated 200,000 persons annually in the U.S., and account for at least 20% of all cancer deaths yearly [4,5]. A major source of brain metastases is adenocarcinoma of the breast [4,5]. Approximately 200,000 women develop breast cancer annually, and 2–3 years after diagnosis nearly 30% develop brain metastases [6,7]. There are few treatment options for this relatively late and lethal complication, which is more frequent in younger patients [8].

A potentially effective therapeutic strategy may derive from the finding that the transmembrane prostate specific membrane antigen (PSMA) is robustly expressed by the tumor vascular endothelium in a variety of solid cancers, including GBM and primary adenocarcinoma of the breast, but is not evident in normal vascular endothelium, and to only very low levels in normal prostate [9-13]. Patient autopsy evidence and preclinical models reveal that progressive gliomas and breast tumor metastases either develop new blood vessels or co-opt existing vessels [14-17]. PSMA has not been detected elsewhere in the body, except on the luminal side of the intestinal epithelium, which is not accessible via the vasculature, and on the apical surface of renal proximal tubular cells, although PSMA targeted antibodies are too large to filter through the kidney glomerulus [18]. Hence, it may be feasible to deliver PSMA targeted agents to the tumor and its microvasculature to (1) selectively destroy the vessels perfusing the tumor tissue, (2) achieve high regional doses of drugs to overcome tumor resistance [19], and (3) spare normal tissues, which typically lack PSMA expression [11]. The vascular endothelium of malignant brain tumors is potentially accessible to a PSMA targeted agent which is important since studies show that the tumor mass may be at least partly protected from therapeutic agents by the blood–brain barrier (BBB) [18,20]. Normal brain parenchyma which expresses low levels of PSMA in a heterogeneous, sparse pattern [21], would be expected to be protected from anti-PSMA targeted antibody agents by an intact BBB [22]. Treatment with cytotoxin-conjugated antibody or nanoparticles coated with a PSMA ligand conjugated to cytotoxins is being actively explored [23,24], and anti-PSMA based agents have targeted and suppressed prostate tumors in preclinical models. Such agents are presently in multiple Phase II clinical trials for prostate tumors and other cancers (http://clinicaltrials.gov/ct2/show/NCT01056029; http://clinicaltrials.gov/show/NCT01695044) [25,26].

A potentially significant reported finding is that breast cancer metastases, while treatment resistant, do not infiltrate surrounding normal brain tissue beyond approximately 1 mm, rather they tend to increase in number and size [27]. This suggests ablation of such metastatic foci and a 1 mm surrounding zone may hold the promise of substantially reducing or eradicating the intracranial tumor burden [27]. PSMA is expressed by the blood vessels of primary breast tumors [9] and importantly, there is an initial report of PSMA expression in breast cancer brain metastases, using manual, general methods of tissue scanning and scoring [28].

For the present study we initially sought to determine, using human tissue samples and fully automated optical scanning and quantification of stained tissue sections, PSMA expression in glioma grades I through IV. We also investigated the intensity and uniformity of PSMA expression by breast cancer brain metastases across patients [9]. The overarching goals of this early feasibility effort was to extend the limited volume of current data using precise automated slide scanning and quantification technology, and to help guide subsequent large scale studies correlating tumor subtypes with patterns of PSMA expression. In addition, given the known important role of PSMA in tumor angiogenesis, further describing its expression may provide a basis for research aimed at understanding; (1) how lower grade gliomas evolve to the highly angiogenic glioblastomas, and (2) how PSMA may contribute to angiogenesis and vasculogenic mimicry in glioblastomas.

## Methods

### Tumor specimens, primary and secondary brain tumors

Under our UCSD IRB approved protocol, 28 Formalin-fixed paraffin-embedded (FFPE) samples were acquired from clinical samples. Tissues included: 4 normal brain samples from autopsies; primary breast cancers and related brain metastases from 4 patients; a total of 14 patient gliomas, including grade I (5 patients), grade II (4 patients), grade III (5 patients) and grade IV (GBM – 5 patients). All glioma samples were acquired before the patients initiated therapy, while the breast tumor metastases were acquired after treatment for the primary tumor, which may be presumed to have been standard although this could not be confirmed for all specimens. One sample of a meningioma was used as a negative staining control for Von Willebrand factor (VWF).

### Sectioning and processing of paraffin imbedded tumors and histology

Each tumor was sectioned at 4um intervals. Paraffin sections were deparaffinized by placing slides in xylene followed by rehydration through graded ethanol (100% - 70%) and washing in phosphate buffered saline-Tween 20 (PBS-T).

### Immunohistochemistry

Deparaffinized and rehydrated sections were placed in target retrieval solution that was heated in a water bath at 96 - 98°C for 30 minutes. In order to quench endogenous peroxidase activity, the sections were incubated for 30 minutes in 0.3% H_2_O_2_ and deionized water. After washing, the sections were incubated for 20 minutes in diluted normal blocking serum, washed with PBS-T, and the primary mouse antibody mAb 3E6 (Dako, Carpenteria, CA) for PSMA was incubated on the sections overnight at 4°C. This antibody has been widely used and has been well validated for staining PSMA in GBM tissue by Wernicke *et al.*[18]. Incubation with the primary antibody was followed by washing and incubation with a biotinylated anti-mouse secondary antibody (RTU Vectastain®), and then application of Vectastain® Elite ABC Kit reagent. Negative controls omitted incubation with the primary antibody but included all other steps. After washing in buffer, the slides were incubated with peroxidase substrate (DAB Peroxidase Substrate Kit). The sections were then counterstained with hematoxylin. Using the same staining protocol, alternating tumor sections were stained with a primary antibody anti VWF murine antibody (Dako, Carpenteria, CA) to detect the microvascular pattern and determine whether PSMA staining corresponded to the blood vessel pattern. A negative staining control was established using adjacent sections of a meningioma in which one was stained with a primary antibody for VWF and underwent the entire staining procedure except that the primary antibody was omitted.

### PSMA staining quantification

The stained sections were scanned using the digital Scanscope® CM-1 scanner and images were subsequently processed using ImageScope® software (Aperio Technology, Vista, CA) with color deconvolution and separation algorithms. All slides were scanned at an absolute magnification of 400× [resolution of 0.25 μm/pixel (100,000 pixels/inch)]. Aperio algorithms make use of a color deconvolution method to separate stains, so that quantification of individual stains avoids cross contamination [29]. Positive Pixel Count (PPC) algorithms calculate the area of positive staining, the average positive intensity (API; optical density), as well as the percentage of weak (1+), medium (2+), and strong (3+) positive staining, relative to a staining calibration curve and normalized to the image mean background intensity.

### Analysis

Staining intensity data were plotted using GraphPad® (La Jolla CA) and analyzed with a one way analysis of variance (ANOVA) and the Wilcoxon-Mann–Whitney test to compare relative PSMA staining intensity in tumors versus normal brain tissues.

## Results

### Expression of PSMA in normal brain

**Figure 1:**
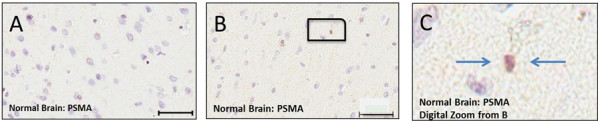
**PSMA staining in tissues samples representative of normal brain. A** – Normal brain showing no PSMA staining. **B** – Normal brain probed for PSMA showing no blood vessel staining and light staining of cellular elements, magnified digitally in **(C)** and indicated by arrows.

In Figure 1, panels A, B and the enlargement in C are representative of the 4 normal patient autopsies and indicate, (1) light staining for PSMA and (2) that the PSMA is only present on a fraction (<1%) of normal brain cells which have been previously reported [21].

### PSMA expression in glioma

**Figure 2:**
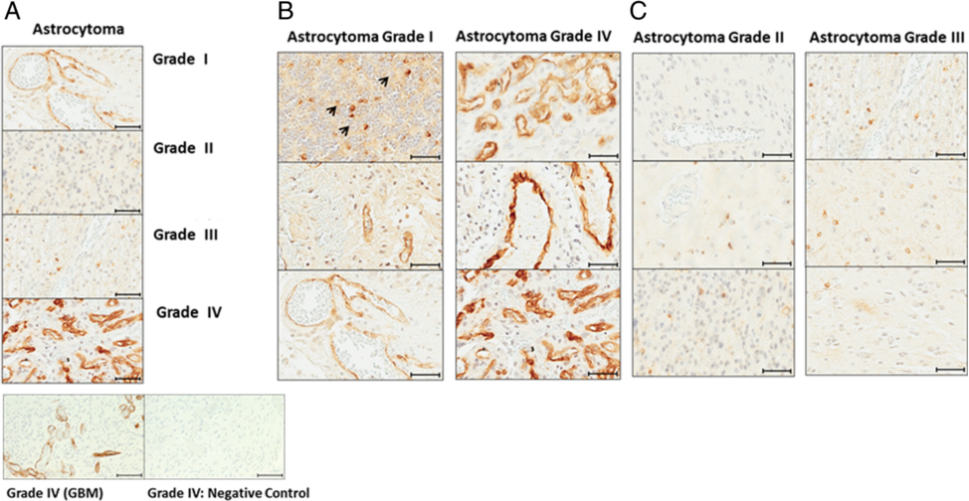
**PSMA staining of grades I – IV glioma tissue Sections. A** - PSMA staining of gliomas grades (I – IV). Note moderate staining of blood vessels and some cells in grade I, only light staining of tumor cells in grades II and III, and heavy blood vessel staining of GBM (grade IV). The bottom two staining control slides show adjacent GBM sections, one stained with the primary antibody for PSMA and the other, the negative control, which was fully stained including the secondary antibody, but with the primary antibody omitted. **B** – PSMA staining for astrocytoma grade I and grade IV (GBM). Each section represents a different patient. Arrows indicate cell staining in grade I tumor tissue Section. **C** – PSMA staining of grade II and III glioma. There is some staining of cells only in grade I and although there is more staining in grade III it is also restricted only to cells, with no blood vessel staining. Size bars in all panels indicate approximately 50 μm, except in negative control sections where they indicate 100 μm.

Figure 2A provides a visual comparison of all four glioma (astrocytoma) grades I through IV, and shows that grade IV glioma blood vessels stained heavily for PSMA, while grades II (n = 4) and III (n = 5) exhibited some staining of tumor parenchyma cells but little vessel staining. In grade I gliomas moderate staining was primarily localized at tumor blood vessels. The two bottom panels of Figure 2A are staining controls. They are adjacent sections from a GBM, one section was stained with a PSMA primary antibody, and the companion section was subjected to the entire staining procedure including the secondary antibody but with the primary antibody omitted (negative control). Figure 2B shows sections from different patients and illustrates that Grade I gliomas (n = 5) did show some tumor cellular staining (arrow) in addition to vessel staining (all panels), and further exploration needs to be pursued to determine the identity of the stained cells. Figure 2B also shows PSMA staining results from individual representative grade IV glioma (GBM) cases, and highlights the heavy concentration of PSMA at tumor microvessels. In contrast Figure 2C, which shows individual cases from grade II and III cases clearly indicates that these gliomas exhibited light staining confined largely to cellular brain elements.

**Figure 3:**
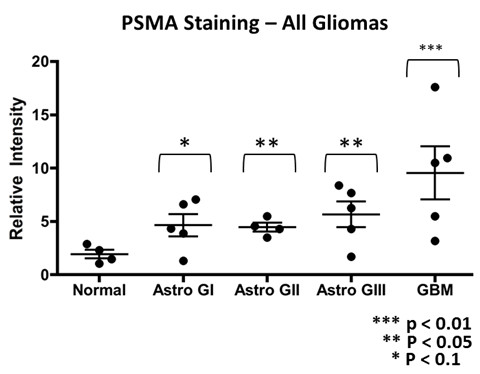
**Summary of PSMA Data.** Graph showing data points, one per patient tumor, and the mean (middle bars) and standard error (outer bars) of PSMA relative staining intensity (normalized to image mean background intensity) for astrocytomas of grades I – IV. Unpaired t–test both parametric and nonparametric forms, p values are indicated.

Quantification of PSMA staining for all types of gliomas relative to a normal brain is summarized in the graph of Figure 3. All categories including normal controls (n = 4) contained individuals with some staining, with the greatest staining intensity displayed by the grade IV (GBM) cases (n = 5) which was on average approximately three times greater than normals. The normal brain surrounding the tumors of glioma grades did not show PSMA staining of the blood vessels. Although the number of patient samples in each of the glioma categories is relatively small, in each case the scanned staining data encompasses entire tissue slides, the staining intensity scale is continuous and finely divided, and the difference between all grades compared with normals is statistically significant. As mentioned previously, grade II and III gliomas were comparable to grade I gliomas in terms of average staining intensity, but in grades II and III the tumor cells but not blood vessels stained, while in grade I staining occurred both in blood vessels and tumor cells.

**Figure 4:**
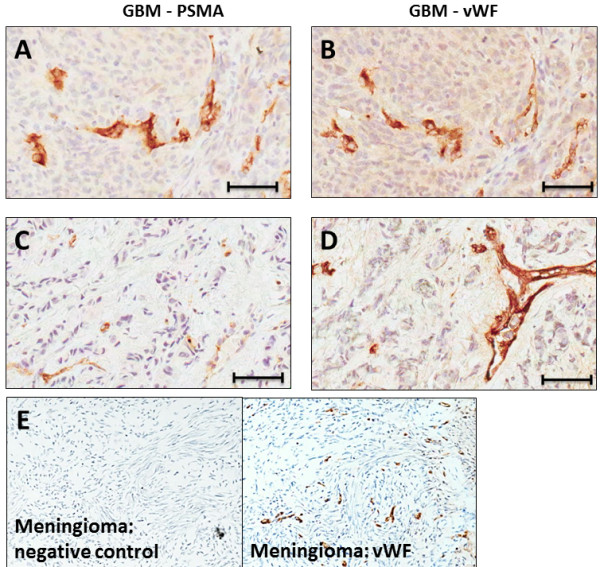
**Correspondence between PSMA and VWF staining in GBM blood vessels. A** and **B -** Adjacent GBM (glioma grade IV) sections stained for PSMA **(A)** and VWF **(B)**. Note that these sections exhibit similar patterns of highlighted blood vessels. The two bottom panels, **(C)** (PSMA) and **(D)** (VWF) exhibit dissimilar staining patterns and tumor cells are stained for PSMA. **(E)** This panel displays two adjacent tissue sections taken for staining control purposes from a meningioma. In the left section the full staining procedure was used except that the primary antibody for VFW was omitted (negative control) and VFW staining is absent, while in the adjacent, right section the primary antibody was included and VFW staining is present. Bars in **A - D** indicate approximately 50 μm.

Staining for VWF and PSMA in adjacent tissue sections revealed a correspondence between PSMA and VWF staining on vascular endothelial cells (Figure 4: panels A and B). Panel C of Figure 4 indicates that other cell types in GBM stained for PSMA, but VWF was largely confined to the tumor microvasculature only (panel D).

### PSMA staining in primary and brain metastatic breast tumors

**Figure 5:**
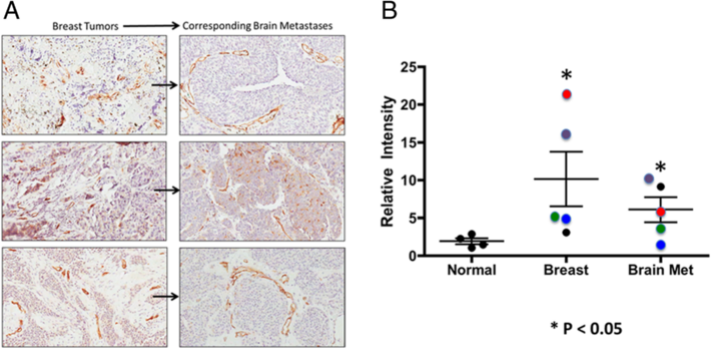
**PSMA Staining in Breast Cancer and Brain Metastases. A** – PSMA staining for breast tumors and breast tumor brain metastases pairs. Each pair of sections is from a different breast tumor case (n = 3). Size bars in all panels indicate approximately 50 μm. **B** – Graph of PSMA staining intensity for primary breast tumors and for breast tumor brain metastases, showing individual patient values and group mean (bars). The color coding indicates those primary breast tumor and brain metastases that came from the same patient (n = 4). Although there was considerable variability, each group included individual PSMA staining data points that were higher than the largest values for normal brain staining in terms of PSMA, these data were significant (p < 0.05), and for 3 of 4 patients staining intensity was less for breast cancer brain metastases than for the primary breast tumors.

Breast cancer tissue exhibited a high degree of PSMA staining that was considerably above that of normal brain (Figure 5A and B). Figure 5A shows color coded matched breast cancer and breast brain cancer metastases (n = 4). Both primary breast cancer and brain metastases had variable staining even within the same tumor. Breast cancer had staining approximately five-fold greater than normals (Figure 5B). Breast cancer brain metastases (n = 5) exhibited staining that was on average approximately threefold greater than that for normal brain (n = 4; p = 0.0007) and in three patients was less than primary breast tumors.

## Discussion

The present study measured the expression of PSMA on human primary and secondary brain cancers to explore the feasibility of PSMA targeting for tumor selective delivery of therapeutic agents. The data indicated that the PSMA is expressed on the brain tumor cells and especially the tumor blood vessels in human gliomas of grades I and IV (Figures 2A & 3). We determined that blood vessels in breast cancer brain metastases exhibit PSMA, although we also found that expression intensity varied considerably between patients (Figure 5A and B). Normal brain tissue from tumor and non-tumor bearing patients had a small number of PSMA positive cells while the microvasculature had no staining (Figure 1). This agrees with a previous report describing cellular PSMA expression according to brain anatomical region [21]. Our results for grade IV gliomas (GBM) align with those of Wernicke *et a*l. [18], and we extend the available data to include grade I to III gliomas. Wernicke *et al.* also reported that PSMA staining was uniform for 14 breast cancer brain metastases and at the same intensity as the primary tumors [28]. We also found PSMA on such metastases, although with considerable variation and in general the staining intensity on metastases was somewhat below that of the matched primary breast tumor. Using automated, complete scanning and quantification to yield a continuous, fine scale, we required relatively few samples to elaborate on Wernicke’s findings.

Despite the fact that primary and secondary brain tumors express high levels of VEGF and are densely vascularized, making anti-angiogenic therapy attractive, VEGF inhibitors have so far been of limited benefit in brain cancer patients but may be potentially be effectively combined with PSMA targeting [17,18,30-33]. The basis for this is that in addition to GBM induction of angiogenesis via VEGF, (1) at least some of the brain tumor vasculature derives from the transdifferentiation of tumor cells and does not depend on VEGF [34-36] and (2) PSMA induces VEGF-independent angiogenesis in pathological conditions [37]. It is also relevant that the stem-like cells driving brain cancers and tumor-derived angiogenesis reside in close proximity to newly formed microvessels, and the expansion of glioma stem like cells in animal models requires a microvascular supply [38,39].

Our observed variability of PSMA staining between tumors of the same type, and the differential staining of grade IV gliomas relative to lower grades needs to be comprehensively investigated in terms of informing and personalizing treatment regimens. All the glioma samples were acquired before the patients were treated, and one explanation for differential staining may be related to previous reports indicating that grade IV gliomas are highly angiogenic compared with lower grades [40], and that PSMA is only expressed on angiogenic vessels [41,42]. This is not entirely unexpected as for prostate cancer evidence suggests that PSMA expression is greatest in high-grade, metastatic, and hormone-insensitive tumors [43]. Another possible basis for differential staining may relate to the observation that the FOLH1 (PSMA) gene generates several splice variants, including PSMA, PSME, PSM’, PSMA-C, PSMA-D, PSMAΔ6, and PSMAΔ18 [44,45]. Grade 1 through IV gliomas which exhibit different behavior and gene signatures, may also have differential expression of FOLH1 splice variants [46]. Moreover, PSMA influences integrin activation, various signaling pathways, and impacts survival and invasion in prostate cancer cells [42]. Since PSMA is expressed by some GBM tumor cells, it would be of potential interest to explore whether different splice variants play a role in the transdifferentiation of GBM cells into vascular elements, *i.e.,* vasculogenic micmicry [47]. The possibility that the expression of FOLH1 splice variants changes according to glioma grade and influences vascular expansion and tumor cell invasion should be investigated in follow-up studies.

The variability in PSMA staining observed with all glioma grades aligns with the phenotypic heterogeneity of gliomas and suggests that; (1) the extent and variability of PSMA staining within glioma grades needs to be further explored in larger scale studies, and (2) tumor PSMA levels should be characterized to better identify patients that would potentially benefit from PSMA targeted therapy. The same concept may apply to breast cancer brain metastases given the heterogeneity that we observed [48].

The present study has expanded on previous data to include all grades of glioma (I through IV). This is important because lower grade gliomas often progress despite treatment and are associated with transformation to more aggressive higher grades [49]. Elaborating on PSMA data for is significant because breast cancer brain metastases represents an emerging, large clinical problem. Even patients with Her-2-positive breast tumors responding well to trastuzumab (Herceptin®) suffer from brain metastasis [8]. It should be noted that the present initial study was constrained by the difficulty in acquiring tissue specimens. A follow-up project will involve other centers to acquire a larger number of patient samples to more accurately quantify the variability of PSMA staining, and importantly, to correlate PSMA levels with different grade IV glioma (GBM) subtypes, *viz*., Proneural, Neural, Classical and Mesenchymal. In addition, PSMA expression will be related to breast cancer subtype.

## Conclusions

This report evaluated PSMA staining in grades I to IV gliomas and breast cancer brain metastases. Average microvessel PSMA levels in grade IV gliomas and breast cancer brain metastases were significantly greater than for normal brain, with variability between samples taken from different patients. These data provide a foundation for further more comprehensive investigations of PSMA in primary and secondary brain tumors; (1) as a target for the selective ablation of tumor microvasculature, (2) as a means of preferentially concentrating therapeutic agents at tumor tissue, and (3) potentially to help understand the progression of low grade gliomas to glioblastoma. Based on this and other data, a prospective clinical trial in glioblastoma is in process (http://clinicaltrials.gov/ct2/show/NCT02067156).

## Abbreviations

ANOVA: Analysis of variance; API: Positive pixel count; BBB: Blood brain barrier; GBM: Glioblastoma (grade IV glioma); PPC: Positive pixel count; PSMA: Prostate specific membrane antigen; VEGF: Vascular endothelial growth factor; VWF: Von Willebrand factor.

## Competing interests

The authors declare that they have no competing interests.

## Authors’ contributions

NN helped formulate study, conducted experiments and data analysis, prepared figures and contributed to writing manuscript. SP had key participation in terms of conducting experiments and in data analysis. PJ contributed to experiments and analysis. GL participated in completion of experiments and provided important feedback. JRC played an important role in data analysis and organization of the manuscript. DP assisted significantly with data interpretation. TJ participated in data interpretation and manuscript preparation. SCP contributed to data analysis and manuscript preparation. MM contributed to data analysis, figure preparation, and writing/editing the manuscript. SK formulated study idea and defined scientific context, examined and analyzed tissue sections, organized data and significantly edited/revised the manuscript. All authors read and approved the final manuscript.

## Authors’ information

SK is a MD, PhD neuro-oncologist with a background in molecular biology. SK’s efforts are divided 50% to treating brain cancer patients and 50% to research. SK leads a multidisciplinary laboratory focused on understanding the developmental and molecular aberrations resulting in malignant gliomas and using this information to develop novel molecular therapeutics to improve patient survival. The laboratory is collaborative, with post-docs, graduate students and project scientists experienced in glioma stem cell biology, signal transduction, brain tumor imaging, genomic and proteomic biomarker discovery, and translational science based on clinical samples from patients at UCSD.
